# Duration and intensity of occupational lifting and risk of long-term sickness absence: Prospective cohort study with register follow-up among 45 000 workers

**DOI:** 10.5271/sjweh.4085

**Published:** 2023-05-01

**Authors:** Rúni Bláfoss, Sebastian Venge Skovlund, Sebastian Skals, Emil Sundstrup, Rubén López-Bueno, Joaquin Calatayud, Lars Louis Andersen

**Affiliations:** 1 National Research Centre for the Working Environment, Copenhagen, Denmark.; 2 Research Unit of Muscle Physiology and Biomechanics, Department of Sports Science and Clinical Biomechanics, University of Southern Denmark, Odense, Denmark.; 3 Department of Physical Medicine and Nursing, University of Zaragoza, Zaragoza, Spain.; 4 Exercise Intervention for Health Research Group (EXINH-RG), Department of Physiotherapy, University of Valencia, Valencia, Spain.; 5 Department of Health Science and Technology, Aalborg University, Aalborg, Denmark.

**Keywords:** absenteeism, aging, musculoskeletal disease, physical workload, public health, occupational exposure, socioeconomic factor

## Abstract

**Objective:**

This study aimed to investigate the prospective association of lifting duration and lifting load with the risk of long-term sickness absence (LTSA).

**Methods:**

We followed manual workers with occupational lifting (N=45 346) from the Work Environment and Health in Denmark Study (2012–2018) for two years in a high-quality national register on social transfer payments (DREAM). Cox regressions with model-assisted weights were employed to estimate the risk of LTSA from lifting duration and loads.

**Results:**

During follow-up, 9.6% of the workers had an episode of LTSA. Compared to workers with seldom lifting (reference), workers lifting ½ and ¾ of the workday had increased risk of LTSA [hazard ratios (HR) of 1.36 [95% confidence interval (CI) 1.20–1.56] and 1.22 (95% CI 1.07–1.39)], respectively. Lifting load showed a positive exposure–response association with LTSA (trend test, P<0.01), with HR for lifting 5–15, 16–29, and ≥30 kg at 1.11 (95% CI 1.02–1.22), 1.17 (95% CI 1.03–1.34), and 1.29 (95% CI 1.11–1.50), respectively. Age-stratified analyses showed increased risk of LTSA among workers ≥50 years with a high proportion of work-related lifting compared to their younger counterparts.

**Conclusions:**

Occupational lifting for ½ the workday increased the risk of LTSA, while higher occupational lifting load exacerbated this risk in an exposure–response manner. The study underscores the importance of reducing both lifting duration and loads for prevention of LTSA at the workplace, especially among older workers.

Physically demanding work, comprising awkward postures, repetitive work, and manual material handling, is a well-established risk factor for sickness absence (1, 2) and remains highly prevalent in modern societies (3). Sickness absence affects individuals, employers, and societies through productivity loss and sickness payment benefits (4, 5). Besides its direct impacts, long-term sickness absence (LTSA) may contribute to workers permanently exiting the labor market before the state pension age (6). Thus, LTSA is associated with both immediate and long-term consequences for work participation. Furthermore, current demographic changes mean that relatively fewer working-age citizens will be available to support the growing number of retirees (7). This results in lower tax incomes to finance the welfare systems and increases the need of formal care due to the growing numbers of senior citizens. Therefore, increasing or retaining work participation is of high societal interest in order to meet the needs of an aging society. To develop effective preventive measures on workplace and societal level it is important to identify work-related risk factors for reduced work participation, eg, LTSA.

High exposure to occupational lifting has previously been suggested as a risk factor for sickness absence (1, 8). Because lifting is a dynamic exposure, which can be quantified in *duration*, *frequency*, and *load*, it is relevant to investigate the importance of different lifting factors in relation to the risk of LTSA. Previous studies investigating work-related physical risk factors for LTSA have assessed lifting load/volume by job exposure matrices (1) and lifting duration at baseline (8). In order to elaborate on existing knowledge, we need large representative studies with sufficient statistical power to investigate the effects of the different aspects of lifting (ie, duration, frequency, and load) on health outcomes (eg, LTSA) and to stratify the analyses by age-group and sex. Thus, large-scale studies investigating the importance of lifting *duration*, *frequency*, and *load* are warranted to support the development of upstream and downstream interventions targeting work participation among workers with physically demanding work. Also it remains to be established whether age- and/or sex-specific differences exist in the risk for LTSA among workers with physically demanding work (9–12). Studies comparing the risks of sickness absence among men and women with physically demanding work have found conflicting results (9, 10), while older workers with physically demanding work seem to be at higher risk of LTSA than their younger comparable counterparts (12). However, these studies investigated physically demanding work in general, and not occupational lifting specifically.

The present prospective cohort study investigated the importance of lifting *duration* and *load* on register-based LTSA among 45 000 representative Danish workers with lifting work. We performed sub-group analyses on age and sex to examine the potential of preventive strategies in these subgroups. We hypothesized that the risk of LTSA would increase in an exposure-response manner with increasing lifting *duration* and *load*, and that older workers and women subjected to these exposures were at higher risk.

## Methods

### Study design and populations

This prospective cohort study with register follow-up is based on the nationwide questionnaire survey Work Environment and Health in Denmark (WEHD) (13, 14). The present study used all four rounds of the questionnaire survey (2012, 2014, 2016, 2018), and linked it to the Danish Register for Evaluation of Marginalisation (DREAM) (12, 15). In each questionnaire round, Statistics Denmark drew a probability sample of Danish citizens between 18–64 years, who worked for a minimum of 35 hours per month and earned ≥3000 DKK (approximately €400) per month for the past three months, and invited them to participate in the study (15). In total, for all four rounds of the survey, Statistics Denmark sent 228 173 invitations and received responses from 127 882 persons (response rate: 56%) (12). The present study included only currently employed wage earners with lifting tasks at work (see definition below in the exposure section) representing most job groups in Denmark and without incident LTSA 52 weeks preceding the questionnaire reply (N=45 346) (12), because sickness absence in the past year predicts future sickness absence (16). In case of participation in several survey rounds, we included only first occasion responses (12). The reporting of this study conforms to the STROBE guidelines for cohort studies (17).

### Occupational lifting (exposure)

We assessed lifting duration and the typical load of the lifting tasks during a working day with two questions. The following question assessed the duration “How much of your working day do you carry or lift?” with the response options in the following order “Almost all the time”, “Approximately ¾ of the time”, “Approximately ½ the time”, “Approximately ¼ of the time”, “Seldom”, or “Never”. As described above, we excluded participants without lifting tasks at work from the analyses, eg, those replying “Never”. We compared lifting duration with the workers answering “Seldom” (reference group). Throughout the manuscript, carrying or lifting is referred to as “lifting”. Lifting load was assessed by the question “What does what you carry or lift typically weigh?”, where the participants had the following response options: “Less than 5 kg”, “5–15 kg”, “16–29 kg”, or “30 kg or more”. We compared lifting load to the group of workers, who typically lifted loads “Less than 5 kg” (reference group).

### Long-term sickness absence (outcome)

Statistics Denmark linked the replies from the WEHD study to the DREAM register using the unique personal identification number (CPR), which all Danish citizens receive at birth or immigration. DREAM contains weekly information about employment and sickness absence payments, and is based on the municipalities’ actual payment information. It is a high-quality register, since the employers have strong economic incentives to receive the compensation (18). In Denmark, the employer covers the first 30 days of sickness absence, while the municipality can compensate the remaining days. The DREAM contains employment and sickness absence on a weekly basis. Therefore, the first day of sickness absence may start in the end of a week, and the last week of sickness absence may end in the beginning of the week. Thus, ≥30 days of sickness absence corresponds to ≥6 weeks in DREAM. We defined LTSA as ≥6 weeks of registered sickness absence for a period of ≤2 years, which started the week after replying the baseline questionnaire (12, 15). We limited the last WEHD round (2018) to comprise a ~1½-year follow-up in order to terminate ultimo 2019, before the COVID-19 pandemic reached Denmark (12, 15).

### Control variables

We adjusted the analyses for the following potential confounders: age (continuous variable), sex (categorical variable: man, woman), year of survey reply (categorical variable: 2012, 2014, 2016, 2018), education (categorical variable: unskilled and skilled workers, further education), use of assistive devices (categorical variable: “always”, “often”, “sometimes”, “seldom”, “never”), leisure-time physical activity (continuous variable: total weekly hours of leisure-time physical activity), body mass index (BMI, kg/m^2^, continuous variable), smoking status (categorical variable: daily, sometimes, ex-smoker, never), psychosocial work factors (continuous variable), and depressive symptoms (continuous variable) (12, 15, 19, 20). Psychosocial work factors were based on the Copenhagen Psychosocial Questionnaire (COPSOQ), which include six items about job strain and two items about influence at work, each converted to a 0–100 scale (0 = worst, 100 = best) (21). Depressive symptoms were based on the Major Depression Inventory (scale 0–50) (22).The chosen control variables may be associated with both exposure and outcome. Because some control variables may function as mediators, which could lead to overadjustment, we present a minimally and a fully adjusted model for all workers. For the stratified analyses, we provide fully adjusted models.

The Central Person Register of Denmark provided age and sex for each individual (12, 15). We also extracted the longest completed education from the register and included in the analyses. The remainder of the control variables were assessed by the questionnaire surveys.

### Statistical analyses

The Cox proportional hazards model (23) (Proc SurveyPhreg of SAS version 9.4) was used to model the hazard ratio (HR) of LTSA during follow-up. Lifting duration and lifting load were the exposure variables. LTSA during follow-up was the outcome in a time-to-first-event analysis. Censoring occurred in the following criteria: reaching the end of the follow-up period, early retirement, disability pension, statutory retirement, emigration, or death. We used model-assisted weights to ensure representative estimates. Model 1 (minimally adjusted) was adjusted for age, sex, education, use of assistive devices, and year of questionnaire reply. Model 2 (fully adjusted) additionally included psychosocial work factors, lifestyle, and depressive symptoms. We also performed stratified analyses of the fully adjusted model (age, sex). Results are reported as HR with 95% confidence intervals (CI).

## Results

Table 1 illustrates the participant characteristics. The study consists of a middle-aged population (mean age: 46 years), equally distributed between sexes and with a mean BMI of 25.9 (slightly overweight). More than 1/5 of the participants smoked daily or occasionally (21.8%), and the mean duration of weekly leisure-time physical activity at either low-, moderate-, or high intensity was 5.2 hours in total. Additionally, during the 2-year follow-up, the weighted incidence of LTSA was 9.6% (95% CI 9.3–9.9%).

**Table 1:** Demographics, lifestyle, and work characteristics. [SD=standard deviation; MDI=Major Depression Inventory.]

		N	%	Mean	SD
Age		45 489		46.2	11.0
Sex		45 489			
	Men	22 501	49.5		
	Women	22 988	50.5		
Questionnaire round		45 489			
	2012	12 060	26.5		
	2014	10 127	22.3		
	2016	11 920	26.2		
	2018	11 382	25.0		
Smoking		44 968			
	Yes, daily	7470	16.6		
	Yes, sometimes	2331	5.2		
	Ex-smoker	12 997	28.9		
	No, never	22 170	49.3		
Body mass index		44 696		25.9	4.5
Leisure-time physical activity (hours/week)		44 971		5.2	3.3
Work environmental factors (0–100)					
	Job strain	45 442		47.1	16.0
	Influence at work	45 405		77.2	19.5
Education		45 142			
	Unskilled and skilled workers	28 508	65.2		
	Further education	16 634	36.9		
Use of assistive devices		45 328			
	Never	11 858	26.2		
	Seldom or sometimes	14 703	32.4		
	Often or always	18 767	41.4		
MDI (0–50)		45 023		8.3	7.5

Table 2 provides the associations of lifting duration and lifting load during work with the risk of register-based LTSA. In the minimally adjusted model (model 1), lifting for ¼ and more of the workday increased the risk of LTSA significantly compared to the reference group of workers (seldom). Workers were at higher risk when lifting for ½ and ¾ of the workday compared to ¼ of the workday, but there were no difference in risk between those lifting for ¾ versus ½ of the workday. In the fully adjusted model, only lifting ½ and ¾ of the workday resulted in elevated risk of LTSA, without a statistically significant difference between lifting ½ or ¾ of the workday.

**Table 2:** Association between lifting duration or lifting load and risk of long-term sickness absence among all workers. [HR=hazard ratio; CI=confidence interval.]

		N		Model 1 ^a^		Model 2 ^b^
				HR (95% CI)		HR (95% CI)
Lifting duration						
	Seldom (reference)	26 373		1		1
	1/4 of the workday	10 809		1.11 (1.01–1.21) ^c^		1.08 (0.98–1.19)
	1/2 the workday	4004		1.43 (1.26–1.63) ^c^		1.36 (1.20–1.56) ^c^
	3/4 of the workday	4160		1.38 (1.21–1.56) ^c^		1.22 (1.07–1.39) ^c^
Lifting load (kg)						
	<5 (reference)	18 174		1		1
	5–15	18 507		1.13 (1.03–1.23) ^c^		1.11 (1.02–1.22) ^c^
	15–29	5309		1.26 (1.10–1.43) ^c^		1.17 (1.03–1.34) ^c^
	≥30	3058		1.46 (1.26–1.70) ^c^		1.29 (1.11–1.50) ^c^

^a^ Adjusted for sex, age, year of questionnaire reply, education, and use of assistive devices. ^b^ Model 1 + body mass index, leisure-time physical activity, smoking, job strain, influence at work, and depressive symptoms. ^c^ Significant difference.

Among all workers, lifting heavier loads at work showed an exposure–response association with increased risk of LTSA (trend test: P<0.01) compared to the workers lifting objects of <5 kg per workday (reference) (table 2). All lifting loads ≥5 kg resulted in statistically significant differences in both models (less and fully adjusted) compared to the reference group.

The fully adjusted model provided slightly lower estimates compared with the minimally adjusted, but the differences were negligible.

Table 3 illustrates the age-stratified analyses in regard to occupational lifting factors and the risk of LTSA. Younger and middle-aged workers (<50 years) had increased risk of LTSA when lifting for ½ of the workday, while older workers (≥50 years) were at an increased risk when lifting for ½ and ¾ of the workday. However, the risk estimates were not higher when lifting ¾ of the workday compared to lifting ½ the workday.

**Table 3:** Association between lifting duration or lifting load and risk of LTSA among all workers stratified by age <50 years and ≥50 years. [HR=hazard ratio; CI=confidence interval.]

		<50 years ^a^			≥50 years ^a^	
		N	HR (95% CI)		N	HR (95% CI)
Lifting duration						
	Seldom (reference)	13 971	1		12 402	1
	1/4 of the workday	6067	1.08 (0.94–1.23)		4742	1.10 (0.96–1.25)
	1/2 the workday	2413	1.37 (1.14–1.63) ^b^		1591	1.40 (1.15–1.69) ^b^
	3/4 of the workday	2715	1.16 (0.98–1.38)		1445	1.37 (1.14–1.66) ^b^
Lifting load (kg)						
	<5 (reference)	9903	1		8271	1
	5–15	10 298	1.09 (0.97–1.23)		8209	1.14 (1.00–1.29) ^b^
	16–29	3111	1.13 (0.94–1.35)		2198	1.24 (1.03–1.50) ^b^
	≥30	1700	1.30 (1.05–1.61) ^b^		1358	1.29 (1.05–1.59) ^b^

^a^ Analyses are adjusted for sex, age, year of questionnaire reply, education, use of assistive devices, BMI, leisure-time physical activity, smoking, job strain, influence at work, and depressive symptoms. ^b^ Significant difference.

For lifting load, workers <50 years had a significantly higher risk of LTSA when lifting objects ≥30 kg. Among workers ≥50 years, a positive exposure–response association existed between all categories of lifting load and the risk of LTSA.

Interaction analyses on age by duration and load did not reach statistical significance (P>0.05). Age by load showed a statistical trend (P=0.0516).

Table 4 shows the sex-stratified analyses. Men reporting lifting ≥¼ of the workday had higher risk of register-based LTSA, whereas women were at higher risk of LTSA when lifting ½ the workday. In terms of lifting load, men had an elevated risk of LTSA when lifting ≥30 kg, while women where at higher risk when lifting 5–15 kg and ≥30 kg.

**Table 4:** Association between lifting duration or lifting load and risk of long-term sickness absence stratified by sex. [HR=hazard ratio; CI=confidence interval.]

		Men ^a^			Women ^a^	
		N	HR (95% CI)		N	HR (95% CI)
Lifting duration						
	Seldom (reference)	12 241	1		14 132	1
	1/4 of the workday	5678	1.18 (1.00–1.38) ^b^		5131	1.03 (0.91–1.16)
	1/2 the workday	2277	1.44 (1.17–1.76) ^b^		1727	1.34 (1.12–1.60) ^b^
	3/4 of the workday	2224	1.30 (1.05–1.62) ^b^		1936	1.17 (0.99–1.39)
Lifting load (kg)						
	<5 (reference)	6888	1		11 286	1
	5–15	9933	1.09 (0.93–1.27)		8574	1.13 (1.01–1.26) ^b^
	16–29	3984	1.15 (0.95–1.38)		1325	1.21 (0.99–1.48)
	≥30	1476	1.29 (1.00–1.67) ^b^		1582	1.28 (1.05–1.55) ^b^

^a^ Analyses are adjusted for sex, age, year of questionnaire reply, education, use of assistive devices, BMI, leisure-time physical activity, smoking, job strain, influence at work, and depressive symptoms. ^b^ Significant difference.

Interaction analyses on sex by duration and load did not reach statistical significance (P>0.05).

## Discussion

The main findings of the present study were that (i) workers reporting lifting at work for ½ and ¾ of the workday had an increased risk of register-based LTSA, and (ii) an exposure–response association existed between higher lifting load and the risk of LTSA. Subgroup analyses indicated that workers ≥50 years had a higher risk of LTSA in terms of both lifting duration and lifting load compared to workers <50 years, while the sex differences were less clear. These results suggest that lifting duration and load are significant risk factors for LTSA among workers with a high degree of occupational lifting. The results add on previous findings, which may function as foundation for upstream and downstream interventions, policy development, and practices for the inspection authorities.

### Interpretation of findings

This study elaborates on previous reports of detrimental effects of occupational lifting in terms of increased risk of sickness absence and early exit from the labor market (1, 8, 9, 24). Somewhat similar to our findings, previous studies among the general working population in Denmark found lifting or carrying at work to increase the risk of register-based LTSA (8, 24). Specifically, Andersen and co-workers found an increased risk of LTSA for persons lifting ≥¼ of the workday (8). However, whereas the latter study reported the effects of lifting duration on LTSA, none of the two studies assessed the prospective association between lifting load and LTSA. Sundstrup et al (1) found number of ton-years (estimated ton lifted per workday for a year) and lifting-years (estimated lifting loads weighing ≥20 kg >10 times per workday for one year) throughout the working life to be associated with increased risks of register-based LTSA in an exposure–response manner. In the present study, lifting heavier loads at work was associated with an increased risk of LTSA compared to workers seldom lifting (reference) in an exposure–response manner, ie, the heavier objects lifted at work, the higher the risk of LTSA (table 2). This exposure–response association indicates that reducing the weight of the loads lifted could potentially prevent futures sickness absence by lowering the risk of LTSA. However, reducing the load of the objects could necessitate an increase in the lifting frequency in order to maintain the same level of production, which may pose another risk factor for LTSA (8, 25). Thus, an optimal strategy to decrease the risk of LTSA could be to increase the use of assistive devices while reducing the load of the objects. Nevertheless, our findings based on a large sample of representative workers indicate that lifting lighter loads would decrease the future risk of LTSA. The present study adds to the current body of knowledge by providing estimates based on a large and representative sample, which allows investigating both different levels of lifting duration and loads as well as stratifying by age and sex.

The present study shows that lifting for ½ and ¾ of the workday increases the risk of LTSA and provide higher risks for LTSA than lifting ¼ of the workday. However, when not adjusting for lifestyle factors, psychosocial work factors, and depressive symptoms (ie, minimally adjusted model), a statistically significant association also existed between lifting for ¼ of the workday and LTSA. Nevertheless, no clear exposure–response association between lifting duration and LTSA existed (tables 2–4). This is somewhat in agreement with a previous study investigating the association between duration of lifting or carrying and register-based LTSA at 2-year follow-up among the general working population in Denmark (8). Here, workers lifting for ≥¼ of the workday were at increased risk of LTSA (8). However, when additionally adjusted for socioeconomic status in the fully adjusted model, lifting ¾ of the workday did not hold a higher risk of LTSA than lifting ½ the workday (8). This latter model may be compared to our study where we only included workers with occupational lifting, ie, most of them may be blue-collar workers. Another difference between the studies is that our sample size is about fourfold the sample size of the referred study, which increases the statistical power and reduces the risk of type II errors. In addition, those lifting frequently may be a selected group of workers (healthy worker effect) (26) who tolerate lifting work, while other workers may have shifted to occupations with less lifting tasks or left the labor market because of poor health. This may result in slightly conservative risk estimates in the present study. Though, the multiple adjustments for potential self-reporting bias factors at baseline in our study reduces the risk of any significant effect on our findings on the association between lifting duration and LTSA.

Our age-stratified analyses shows that workers ≥50 years are at increased risk of LTSA when lifting for ½ and ¾ of the workday as well as an exposure–response association between lifting load (≥5 kg) and LTSA, while workers <50 years are at increased risk when lifting for ½ the workday and ≥30 kg. This is in accordance with a recent study where workers ≥50 years from the general working population in Denmark had higher risks of register-based LTSA due to high physical work demands compared to their comparably younger counterparts (<50 years) (12). Because the present research project aimed to investigate differences in age, we provided age-stratified analyses although the interaction analyses did not reach statistical significance. In the present study, both age groups had an increased risk of LTSA when lifting for ½ the workday, whereas the workers ≥50 years were also at increased risk when lifting ¾ of the workday. The explanation for the latter finding can be due to misclassification bias as discussed in the limitations and strengths section below. Musculoskeletal pain could be one mechanistic pathway through which high exposure to occupational lifting increases the risk of musculoskeletal pain (27, 28), which in turn could make the physical work even more difficult to handle (29, 30). This may result in an imbalance between the worker’s physical capacity and the physical demands of the work, which for some would (at a later time point) reduce work ability and increase the risk of sickness absence in the future (31). Another reason for the higher consequences for the older workers can be the age-related decrease in physical capacity due to decreased activity level and biological factors (32, 33). A decrease in physical capacity with aging would result in relatively higher physical work demands, eventually approaching the physical capacity threshold where the physical work demands surpass the physical capacity of the worker (34, 35), consequently entailing an increased risk of future sickness absence (31).

The sex-stratified analyses (table 4) showed less clear associations compared to the age- and non-stratified analyses. This may seem somewhat surprising, as men have higher physical capacity than women (36, 37). However, equivocal findings exist on whether physical work environment exposures have a more detrimental effect on women than men (9–12). In addition, our interaction analyses on sex by lifting duration and load did not reach statistical significance. Because this project aimed to investigate differences in sex, we provided sex-stratified results. Although recent studies do not observe differences between sexes in the deterioration of the physical capacity with increasing age, men have a higher physical capacity than women throughout the lifespan (35, 38, 39), which some refer to as a “female disadvantage” (39). This difference in the physical capacity between the sexes should be kept in mind when organizing physically demanding work comprising lifting tasks in order to prevent sickness absence. In the present study, a lack of statistical power due to lower exposure to lifting among women may be one explanation for the lack of significant associations. Table 4 visualizes this hypothesis with several borderline significant estimates. Thus, we should interpret the sex-stratified analyses with caution.

### Practical applications

Due to the large sample size, this study serves quite detailed information about where to target interventions to prevent LTSA. The increased risk of LTSA with increased lifting load illustrates that reducing the weight of the objects lifted could reduce the risk of future LTSA. Besides reducing the lifting load, it is important to be aware of the total duration of lifting during the working day. In particular, workplaces should have older workers (≥50 years) in mind when organizing their work, because they seem at increased risk of LTSA. Furthermore, our findings could serve as a future foundation for a potential update in the lifting thresholds that the inspection authorities follow. In addition, the results may instigate initiatives for giving workplaces better opportunities and support to acquire technical assistive devices as well as counselling on how to re-organize the work to secure adequate resting periods, and perhaps increase the demands of using assistive devices. At a higher political level, an increased focus on offering part-time sickness absence could be a priority, because part-time sickness absence shows promising results in earlier recovery to full work capacity, for example among workers on sickness absence due to musculoskeletal disorders (40). An effective strategy for reducing total lifting load is to use assistive devices (41, 42), which potentially could reduce the risk of LTSA – particularly among workers ≥50 years. Besides reducing lifting load, proper use of assistive devices would reduce lifting duration as well. Another initiative could be to re-organize the work and secure adequate rest periods during the workday, which a recent feasibility study found to be potent for reducing pain and fatigue as well as increasing self-rated energy level (43). A strategy could also be to increase the physical capacity and thereby decrease the workers’ relative exposure during occupational lifting tasks. Previous studies have shown that brief periods of strength training at the workplace could increase muscular strength in parallel with reducing musculoskeletal pain among workers with physically demanding work (44). Additionally, performing micro-exercises (short and simple exercise sessions) at the workplaces may potentially reduce future LTSA by 13% among the general working population (45).

### Limitations and strengths

The self-reported exposures might lead to a certain degree of misclassification bias, where the workers may have difficulties rating the degree of both the duration of lifting and the lifting load (46). It may be easier for the workers to rate the weight of the typical objects they manually handle than it is to estimate the time spent lifting during the workday, hence the clearer exposure–response association between lifting load and LTSA. Second, information from questionnaires on physical behaviors is prone to systematic bias by for example musculoskeletal disorders, and socioeconomic and demographical variables (47, 48). In addition, the self-reporting may be prone to recall bias and other potential biases (49, 50). These biases may blur the associations to some extent. However, the large sample and the model-assisted weights contribute to achieving representative estimates. Additionally, the healthy worker effect could lead to confounding bias, which could result in slightly conservative risk estimates in the present study. Third, DREAM does not contain information on the cause of sickness absence, and, therefore, the participants’ sickness absence may originate from causes not mainly affected by occupational lifting. Fourth, an explanation for the statistical differences in the age-stratified analyses might be due to statistical power, where more workers <50 years are lifting for ½ and ¾ of the workday than workers ≥50 years, and the lower CI is close to being statistically significant among workers <50 years lifting ¾ of the workday. Fifth, because lifting is a dynamic exposure divided into *duration, frequency*, and *load*, it is a limitation of the study that our data only contains information on lifting duration and load, and not frequency. Furthermore, the survey item of the exposure “lifting duration” also comprises carrying. Carrying can include statically lifting while walking, while it could also impose a different exposure than the more dynamic lifting motion. Sixth, the present dataset does not contain information on job seniority, which could affect the risk estimates. However, people rarely switch from physically demanding jobs to sedentary jobs, and vice versa. In the WEHD study, some people participated in more than one questionnaire round between 2012 and 2018. Using these data and a previously published ‘ergonomic index’ (51), we can see that individual physical work demands are very stable over a two-year period (Spearman correlation coefficient: 0.83 (P<0.0001), mean difference -0.1 on a scale of 0–100, N=32 314). Finally, because the duration of follow-up is up to two years maximum, this study might not reflect all detrimental effects of both duration and intensity of occupational lifting on the risk for LTSA in the working life, which may be higher than observed in the present study.

The use of model-assisted weights on a random sample of the general working population in Denmark strengthens the study by making the estimates representative for workers in Denmark exposed to occupational lifting. DREAM is considered to be highly accurate due to the economic incentive of the employers to correctly report and, hereby, receive adequate sickness absence compensation (18). The large sample size of the general working population in Denmark with lifting work allows for investigating exposure–response associations, stratifying by age and sex, and generalizing the results to the Danish working population with lifting tasks at work. Lastly, we performed post hoc sensitivity analyses to investigate the potential effect of working hours, where we (i) adjusted for working hours and (ii) only included those working 30–45 hours per week. This did not change the overall results, and only lead to second-decimal changes in the HR.

### Concluding remarks

Performing lifting tasks ≥½ the working day increased the risk of LTSA during a 2-year register follow-up, while higher lifting load increased the risk of LTSA in an exposure–response manner. Workers ≥50 years were generally at increased risk of LTSA, while the sex-differences in the sex-stratified analyses were less clear. The study underscores the importance of prioritizing the physical working environment for preventing LTSA at the workplace, eg, by reducing both lifting duration and loads.

## Data Availability

The authors encourage collaboration and use of the data by other researchers. Data is stored on the secure server of Statistics Denmark, and researchers interested in using the data for scientific purposes should contact Professor Lars Louis Andersen ( lla@nfa.dk).

## Protection of research participants

All data are stored on the secure server of Statistics Denmark and only available in anonymized format to the researchers (52).

## References

[1] Sundstrup E, Hansen ÃM, Mortensen EL, Poulsen OM, Clausen T, Rugulies R et al. Cumulative occupational mechanical exposures during working life and risk of sickness absence and disability pension: prospective cohort study. Scand J Work Environ Health 2017 Sep;43(5):415–25. 10.5271/sjweh.3663 28783203

[2] Pedersen J, Bjorner JB, Andersen LL. Physical work demands and expected labor market affiliation (ELMA): prospective cohort with register-follow-up among 46 169 employees. Scand J Work Environ Health 2022 Nov;48(8):641–50. 10.5271/sjweh.4050 35789276 PMC10546615

[3] Hulshof CT, Pega F, Neupane S, van der Molen HF, Colosio C, Daams JG et al. The prevalence of occupational exposure to ergonomic risk factors: A systematic review and meta-analysis from the WHO/ILO Joint Estimates of the Work-related Burden of Disease and Injury. Environ Int 2021 Jan;146:106157. 10.1016/j.envint.2020.106157 33395953

[4] Bevan S. Economic impact of musculoskeletal disorders (MSDs) on work in Europe. Best Pract Res Clin Rheumatol 2015 Jun;29(3):356–73. 10.1016/j.berh.2015.08.002 26612235

[5] van der Wurf C, Speklé E, Schaafsma F, Coenen P. Determining the Costs of Low-Back Pain Associated Sick Leave in the Dutch Workforce in the Period 2015 to 2017. J Occup Environ Med 2021 Jun;63(6):e367–72. 10.1097/JOM.0000000000002221 34048385

[6] Helgadóttir B, Narusyte J, Ropponen A, Bergström G, Mather L, Blom V et al. The role of occupational class on the association between sickness absence and disability pension: A Swedish register-based twin study. Scand J Work Environ Health 2019 Nov;45(6):622–30. 10.5271/sjweh.3816 30891611

[7] Kiss M. Demographic Outlook for the European Union - 2022 [Internet]. European Parliament; 2022 May [cited 2023 May 1]. Available from: https://www.europarl.europa.eu/RegData/etudes/STUD/2022/729461/EPRS_STU(2022)729461_EN.pdf

[8] Andersen LL, Fallentin N, Thorsen SV, Holtermann A. Physical workload and risk of long-term sickness absence in the general working population and among blue-collar workers: prospective cohort study with register follow-up. Occup Environ Med 2016 Apr;73(4):246–53. 10.1136/oemed-2015-103314 26740688

[9] Thorsen SV, Flyvholm MA, Pedersen J, Bültmann U, Andersen LL, Bjorner JB. Associations between physical and psychosocial work environment factors and sickness absence incidence depend on the lengths of the sickness absence episodes: a prospective study of 27 678 Danish employees. Occup Environ Med 2021 Jan;78(1):46–53. 10.1136/oemed-2020-106554 32907881 PMC7803903

[10] d’Errico A, Costa G. Socio-demographic and work-related risk factors for medium- and long-term sickness absence among Italian workers. Eur J Public Health 2012 Oct;22(5):683–8. 10.1093/eurpub/ckr140 22158884

[11] Ervasti J, Pietiläinen O, Rahkonen O, Lahelma E, Kouvonen A, Lallukka T et al. Long-term exposure to heavy physical work, disability pension due to musculoskeletal disorders and all-cause mortality: 20-year follow-up-introducing Helsinki Health Study job exposure matrix. Int Arch Occup Environ Health 2019 Apr;92(3):337–45. 10.1007/s00420-018-1393-5 30511342 PMC6420465

[12] Andersen LL, Pedersen J, Sundstrup E, Thorsen SV, Rugulies R. High physical work demands have worse consequences for older workers: prospective study of long-term sickness absence among 69 117 employees. Occup Environ Med 2021 Nov;78(11):829–34. 10.1136/oemed-2020-107281 33972376 PMC8526881

[13] Andersen LL, Thorsen SV, Flyvholm MA, Holtermann A. Long-term sickness absence from combined factors related to physical work demands: prospective cohort study. Eur J Public Health 2018 Oct;28(5):824–9. 10.1093/eurpub/cky073 29741617 PMC6148972

[14] Johnsen NF, Thomsen BL, Hansen JV, Christensen BS, Rugulies R, Schlünssen V. Job type and other socio-demographic factors associated with participation in a national, cross-sectional study of Danish employees. BMJ Open 2019 Aug;9(8):e027056. 10.1136/bmjopen-2018-027056 31427315 PMC6701570

[15] Andersen LL, Vinstrup J, Thorsen SV, Pedersen J, Sundstrup E, Rugulies R. Combined psychosocial work factors and risk of long-term sickness absence in the general working population: prospective cohort with register follow-up among 69 371 workers. Scand J Work Environ Health 2022 Sep;48(7):549–59. 10.5271/sjweh.4035 35647686 PMC10539106

[16] Roelen CA, Koopmans PC, Schreuder JA, Anema JR, van der Beek AJ. The history of registered sickness absence predicts future sickness absence. Occup Med (Lond) 2011 Mar;61(2):96–101. 10.1093/occmed/kqq181 21173042

[17] von Elm E, Altman DG, Egger M, Pocock SJ, Gøtzsche PC, Vandenbroucke JP; STROBE Initiative. The Strengthening the Reporting of Observational Studies in Epidemiology (STROBE) statement: guidelines for reporting observational studies. J Clin Epidemiol 2008 Apr;61(4):344–9. 10.1016/j.jclinepi.2007.11.008 18313558

[18] Hjollund NH, Larsen FB, Andersen JH. Register-based follow-up of social benefits and other transfer payments: accuracy and degree of completeness in a Danish interdepartmental administrative database compared with a population-based survey. Scand J Public Health 2007;35(5):497–502. 10.1080/14034940701271882 17852980

[19] Virtanen M, Ervasti J, Head J, Oksanen T, Salo P, Pentti J et al. Lifestyle factors and risk of sickness absence from work: a multicohort study. Lancet Public Health 2018 Nov;3(11):e545–54. 10.1016/S2468-2667(18)30201-9 30409406 PMC6220357

[20] Thern E, Falkstedt D, Almroth M, Kjellberg K, Landberg J, Bodin T et al. Educational qualification differences and early labor market exit among men: the contribution of labor market marginalization measured across the working life. BMC Public Health 2022 May;22(1):1015. 10.1186/s12889-022-13397-1 35590290 PMC9121573

[21] Pejtersen JH, Kristensen TS, Borg V, Bjorner JB. The second version of the Copenhagen Psychosocial Questionnaire. Scand J Public Health 2010 Feb;38(3 Suppl):8–24. 10.1177/1403494809349858 21172767

[22] Bech P, Rasmussen NA, Olsen LR, Noerholm V, Abildgaard W. The sensitivity and specificity of the Major Depression Inventory, using the Present State Examination as the index of diagnostic validity. J Affect Disord 2001 Oct;66(2-3):159–64. 10.1016/S0165-0327(00)00309-8 11578668

[23] Cox DR. Regression Models and Life Tables. J R Stat Soc B 1972;34:187–220. 10.1111/j.2517-6161.1972.tb00899.x

[24] Lund T, Labriola M, Christensen KB, Bültmann U, Villadsen E. Physical work environment risk factors for long term sickness absence: prospective findings among a cohort of 5357 employees in Denmark. BMJ 2006 Feb;332(7539):449–52. 10.1136/bmj.38731.622975.3A 16446280 PMC1382535

[25] Sundstrup E, Hansen ÅM, Mortensen EL, Poulsen OM, Clausen T, Rugulies R et al. Retrospectively assessed physical work environment during working life and risk of sickness absence and labour market exit among older workers. Occup Environ Med 2018 Feb;75(2):114–23. 10.1136/oemed-2016-104279 28819019 PMC5800344

[26] McMichael AJ. Standardized mortality ratios and the “healthy worker effect”: scratching beneath the surface. J Occup Med 1976 Mar;18(3):165–8. 10.1097/00043764-197603000-00009 1255276

[27] Coenen P, Gouttebarge V, van der Burght AS, van Dieën JH, Frings-Dresen MH, van der Beek AJ et al. The effect of lifting during work on low back pain: a health impact assessment based on a meta-analysis. Occup Environ Med 2014 Dec;71(12):871–7. 10.1136/oemed-2014-102346 25165395

[28] Coenen P, Kingma I, Boot CR, Bongers PM, van Dieën JH. Cumulative mechanical low-back load at work is a determinant of low-back pain. Occup Environ Med 2014 May;71(5):332–7. 10.1136/oemed-2013-101862 24676271

[29] Skovlund SV, Bláfoss R, Sundstrup E, Andersen LL. Association between physical work demands and work ability in workers with musculoskeletal pain: cross-sectional study. BMC Musculoskelet Disord 2020 Mar;21(1):166. 10.1186/s12891-020-03191-8 32171283 PMC7071559

[30] Ezzatvar Y, Calatayud J, Andersen LL, Vinstrup J, Alarcón J, Casaña J. Dose-response association between multi-site musculoskeletal pain and work ability in physical therapists: a cross-sectional study. Int Arch Occup Environ Health 2020 Oct;93(7):863–70. 10.1007/s00420-020-01533-6 32206864

[31] Kinnunen U, Nätti J. Work ability score and future work ability as predictors of register-based disability pension and long-term sickness absence: A three-year follow-up study. Scand J Public Health 2018 May;46(3):321–30. 10.1177/1403494817745190 29212430

[32] Troiano RP, Berrigan D, Dodd KW, Mâsse LC, Tilert T, McDowell M. Physical activity in the United States measured by accelerometer. Med Sci Sports Exerc 2008 Jan;40(1):181–8. 10.1249/mss.0b013e31815a51b3 18091006

[33] Walston JD. Sarcopenia in older adults. Curr Opin Rheumatol 2012 Nov;24(6):623–7. 10.1097/BOR.0b013e328358d59b 22955023 PMC4066461

[34] Peeters G, Dobson AJ, Deeg DJ, Brown WJ. A life-course perspective on physical functioning in women. Bull World Health Organ 2013 Sep;91(9):661–70. 10.2471/BLT.13.123075 24101782 PMC3790225

[35] Skelton DA, Mavroeidi A. How do muscle and bone strengthening and balance activities (MBSBA) vary across the life course, and are there particular ages where MBSBA are most important? J Frailty Sarcopenia Falls 2018 Jun;3(2):74–84. 10.22540/JFSF-03-074 32300696 PMC7155320

[36] Lewis DA, Kamon E, Hodgson JL. Physiological differences between genders. Implications for sports conditioning. Sports Med 1986;3(5):357–69. 10.2165/00007256-198603050-00005 3529284

[37] Sandbakk Ø, Solli GS, Holmberg HC. Sex Differences in World-Record Performance: The Influence of Sport Discipline and Competition Duration. Int J Sports Physiol Perform 2018 Jan;13(1):2–8. 10.1123/ijspp.2017-0196 28488921

[38] Suetta C, Haddock B, Alcazar J, Noerst T, Hansen OM, Ludvig H et al. The Copenhagen Sarcopenia Study: lean mass, strength, power, and physical function in a Danish cohort aged 20-93 years. J Cachexia Sarcopenia Muscle 2019 Dec;10(6):1316–29. 10.1002/jcsm.12477 31419087 PMC6903448

[39] Sialino LD, Schaap LA, van Oostrom SH, Nooyens AC, Picavet HS, Twisk JW et al. Sex differences in physical performance by age, educational level, ethnic groups and birth cohort: The Longitudinal Aging Study Amsterdam. PLoS One 2019 Dec;14(12):e0226342. 10.1371/journal.pone.0226342 31851709 PMC6919600

[40] Andrén D, Svensson M. Part-time sick leave as a treatment method for individuals with musculoskeletal disorders. J Occup Rehabil 2012 Sep;22(3):418–26. 10.1007/s10926-011-9348-7 22270230

[41] Vinstrup J, Jakobsen MD, Madeleine P, Andersen LL. Physical exposure during patient transfer and risk of back injury & low-back pain: prospective cohort study. BMC Musculoskelet Disord 2020 Oct;21(1):715. 10.1186/s12891-020-03731-2 33129282 PMC7603727

[42] Januario LB, Mathiassen SE, Stevens ML, Holtermann A, Bergström G, Rugulies R et al. Are resident handlings in eldercare wards associated with musculoskeletal pain and sickness absence among the workers? A prospective study based on onsite observations. Scand J Work Environ Health 2021 Nov;47(8):609–18. 10.5271/sjweh.3979 34397097 PMC9058617

[43] Lerche AF, Mathiassen SE, Rasmussen CL, Straker L, Søgaard K, Holtermann A. Development and Implementation of ‘Just Right’ Physical Behavior in Industrial Work Based on the Goldilocks Work Principle-A Feasibility Study. Int J Environ Res Public Health 2021 Apr;18(9):4707. 10.3390/ijerph18094707 33925078 PMC8125316

[44] Sundstrup E, Seeberg KG, Bengtsen E, Andersen LL. A Systematic Review of Workplace Interventions to Rehabilitate Musculoskeletal Disorders Among Employees with Physical Demanding Work. J Occup Rehabil 2020 Dec;30(4):588–612. 10.1007/s10926-020-09879-x 32219688 PMC7716934

[45] Andersen LL, Skovlund SV, Vinstrup J, Geisle N, Sørensen SI, Thorsen SV et al. Potential of micro-exercise to prevent long-term sickness absence in the general working population: prospective cohort study with register follow-up. Sci Rep 2022 Feb;12(1):2280. 10.1038/s41598-022-06283-8 35145176 PMC8831624

[46] Viikari-Juntura E, Rauas S, Martikainen R, Kuosma E, Riihimäki H, Takala EP et al. Validity of self-reported physical work load in epidemiologic studies on musculoskeletal disorders. Scand J Work Environ Health 1996 Aug;22(4):251–9. 10.5271/sjweh.139 8881013

[47] Wiktorin C, Karlqvist L, Winkel J; Stockholm MUSIC I Study Group. Validity of self-reported exposures to work postures and manual materials handling. Scand J Work Environ Health 1993 Jun;19(3):208–14. 10.5271/sjweh.1481 8367699

[48] Sabia S, van Hees VT, Shipley MJ, Trenell MI, Hagger-Johnson G, Elbaz A et al. Association between questionnaire- and accelerometer-assessed physical activity: the role of sociodemographic factors. Am J Epidemiol 2014 Mar;179(6):781–90. 10.1093/aje/kwt330 24500862 PMC3939851

[49] Podsakoff PM, MacKenzie SB, Lee JY, Podsakoff NP. Common method biases in behavioral research: a critical review of the literature and recommended remedies. J Appl Psychol 2003 Oct;88(5):879–903. 10.1037/0021-9010.88.5.879 14516251

[50] Choi BC, Pak AW. A catalog of biases in questionnaires. Prev Chronic Dis 2005 Jan;2(1):A13. 15670466 PMC1323316

[51] Andersen LL, Vinstrup J, Sundstrup E, Skovlund SV, Villadsen E, Thorsen SV. Combined ergonomic exposures and development of musculoskeletal pain in the general working population: A prospective cohort study. Scand J Work Environ Health 2021 May;47(4):287–95. 10.5271/sjweh.3954 33749799 PMC8091072

[52] Danmarks Statistik. Databeskyttelse af personoplysninger [Internet]. [Statistics Denmark. Data protection of personal data]. 2022. Available from: https://www.dst.dk/da/Indberet/vilkaar-for-indberetning/beskyttelse-af-persondata

